# Spatiotemporal heterogeneity of social contact patterns related to infectious diseases in the Guangdong Province, China

**DOI:** 10.1038/s41598-020-63383-z

**Published:** 2020-04-15

**Authors:** Yulin Huang, Xiaoshuang Cai, Bing Zhang, Guanghu Zhu, Tao Liu, Pi Guo, Jianpeng Xiao, Xing Li, Weilin Zeng, Jianxiong Hu, Wenjun Ma

**Affiliations:** 1 The First Affiliated Hospital of Jinan University, Guangzhou, China; 2 Guangdong Provincial Institute of Public Health, Guangdong Provincial Center for Disease Control and Prevention, Guangzhou, China; 3 School of Basic Medicine, Jinan University, Guangzhou, China; 4 Medical College, Shantou University, Shantou, China

**Keywords:** Infectious diseases, Disease prevention, Epidemiology, Population screening, Risk factors

## Abstract

The social contact patterns associated with the infectious disease transmitted by airborne droplets or close contact follow specific rules. Understanding these processes can improve the accuracy of disease transmission models, permitting their integration into model simulations. In this study, we performed a large-scale population-based survey to collect social contact patterns in three cities on the Pearl River Delta of China in winter and summer. A total of 5,818 participants were face-to-face interviewed and 35,542 contacts were recorded. The average number of contacts per person each day was 16.7 considering supplementary professional contacts (SPCs). Contacts that occurred on a daily basis, lasted more than 4 hours, and took place in households were more likely to involve physical contact. The seasonal characteristics of social contact were heterogeneous, such that contact in the winter was more likely to involve physical contact compared to summer months. The spatial characteristics of the contacts were similar. Social mixing patterns differed according to age, but all ages maintained regular contact with their peers. Taken together, these findings describe the spatiotemporal distribution of social contact patterns relevant to infections in the Guangdong Province of China. This information provides important parameters for mathematical models of infectious diseases.

## Introduction

Many of the infectious diseases transmitted by airborne droplets or close contact are spread from person to person. Acquiring the authentic parameters of contact patterns is critical to improve the accuracy of mathematical models to predict the spread of infections and to assess preventive measures. Currently, the measurement of contact patterns mainly includes direct observations, contact diaries and proximity sensors. Using these methods, valuable contacts parameters, including duration and frequency, can be calculated. Social contact patterns have been reported in Europe^1–6^, China^7–9^, Japan^10^, Vietnam^11^, Peru^12^, Kenya^13^ and other regions^14–21^. The development of mathematical models incorporating these patterns according to region can capture the transmission modes of some diseases, including mumps, influenza, varicella, parvovirus, and hand-foot-and-mouth disease^6,22–24^. These data help to clarify risk factors and improve interventions. For example, the famous POLYMOD study^1^, which investigated the social contact patterns in eight European countries combined with serological data found that intimate contact can explain the transmission of varicella and parvovirus B19 infection^24^. Previous contact studies have mainly investigated differences between weekdays, weekends and during school closures^25,26^, whilst surveys aiming to explore seasonal differences are lacking. Seasonal changes in contact patterns between susceptible populations and infected individuals are often considered an important driver of seasonality in infectious diseases such as influenza^27,28^.

China, a country that occupies one-seventh of the world’s population, plays an important role in global pandemics of respiratory-transmitted diseases such as influenza. Studies on social contact pattern have been carried out in China previously, but large-scale survey is still lack for the moment. The Guangdong Province is in South China and had a well-developed economy and trade, a high population density and high intensity connections across the globe, with a far-reaching impact on global pandemics of respiratory infectious diseases. Hence, we conducted a large-scale social contact survey in different seasons across different city in the Guangdong province to obtain important parameters for mathematical modeling of infectious diseases based on the Chinese population and to identify spatiotemporal heterogeneity of social contact patterns.

## Materials and methods

### Survey respondents

We performed a population-based cross-sectional study in the Pearl River Delta of Guangdong Province. Multistage cluster random sampling methods were used to recruit and enroll participants. The Yuexiu, Conghua and Panyu Districts in Guangzhou City, the Chancheng and Sanshui District in Foshan City, and the Doumen District in Zhuhai City were randomly selected as survey sites following standard protocols. In each district, a community was randomly selected from communities with a population of at least 10,000 residents. All households in each selected community were numbered and systematic sampling was applied to households. All family members aged >6 months of each selected household were enrolled for survey assessments and each district had a sample size of ~1,000. A total of 5858 (1902 households) participants were investigated. We compared the age structure between census data (2015) and sample data of the Guangdong Province as shown in Supplementary Table S1.

### Survey contents and methods

The survey was performed by trained interviewers through face-to-face interviews during the winter (December, January and February) and summer (June, July and August) of 2016. The definition of contact was a conversation with three or more words or physical contacts including handshakes, hugs, kisses, and ball games. The questionnaires included basic information of the respondents and contact information from the previous day, based on the POLYMOD study^1^. The basic information consisted of age, gender, household size, occupation, residential address, and season. Details on contact (physical contact or not), the frequency of contact (almost every day, once or twice per week, once or twice per month, less than once per month, upon first meet), the location of contact (home, school, office, transportation, leisure areas, other areas), the duration of contact (less than 5 min, 5 to 15 mins, 15 to 60 mins, 1 to 4 hours, more than 4 hours) and the relationship with participants (relatives, colleagues/classmates, friends and others) were obtained. If participants met some person several times one day, it would be recorded just one item with recording the total duration.

### Professional contacts

In our study, participants could record 12 contacts with detailed contact information. When there were more than 12 contacts per day for a participant, the total number of contacts were recorded as supplementary professional contacts (SPCs). Unless mentioned, the results concerning the model and contact matrix were not included SPC (see Text S1 for more details).

### Dropout rates and missing values

Based on our sampling process, a total of 1902 households were recruited, and all family members of each household were interviewed after informed consent. We choose neighbors as substitutes adjacent to the selected participants who were unable for interview. Data from 40 participants (0.7%) were omitted from our analysis due to missing data.

### Data analysis

We first described the distribution of the contact numbers amongst the age groups. A Generalized Additive Model (GAM) with a negative binomial distribution was used to analyze the association between the number of contacts, including SPCs with the selected variables (age, sex, season survey, household size and occupation), which compares the influence of the variables on the number of contacts including SPCs. Chi-squared tests were used to compare the distribution of contacted individuals by contact features (relationship, location, frequency, duration of contacts) between summer and winter months and amongst the three cities. Differences were considered statistically significant at *P* < 0.05.

Age-related social contact patterns were displayed as the mean number of contacts *c*_*ij*_. The *i* and *j* in *c*_*ij*_ referred to the age groups and contacts of the participants, respectively, including *i*, *j* = 1, 2…,10, consistent with 0–4, 5–9, 10–14, 15–19, 20–29, 30–39, 40–49, 50–59, 60–69 and 70+ years, respectively. The formula for the mean number of contacts was ${c}_{ij}={T}_{ij}/{N}_{i}$, where *T*_*ij*_ = the total contact number in each age group *i* relevant to contacts in the age group *j*, and *N*_*i*_ represents the number of participants in each age group *i*. Sampling weights for each age group were calculated based on official census data of the year 2015 (see Table S1), and used to correctly estimate the mean number of contacts.

Data analysis were performed on R.3.4.0 software (R package *mgcv* and *social mixr*^1^). All figures were plotted using R package *ggplot2*.

## Results

### Characteristics of the respondents

We collected data from 5,818 participants (Table 1). In total, 3,026 (52.0%) participants were females and 1,062 (18.3%) were under 19 years old. The mean age of respondents was 40.7 (SD, 21.3) years and the mean size of each household was 4.0 (SD, 1.3). Of the participants, 15.1% (878) were students and 46.9% (2,729) were employed.

**Table 1:** Number of Contacts including SPCs per Participant per Day according to Characteristics and Relative Number of Contacts from the Generalized Additive Model.

Category	Covariate	Number of Participants	Mean(SD) of Number of Reported Contacts	Reported Contacts	
				Relative Number	95%CI
Age (y)	0−	301	17.8(15.1)	1.00	
	5−	310	29.3(17.4)	1.53	1.32, 1.77
	10−	246	27.7(19.8)	1.43	1.22, 1.68
	15−	205	25.2(17.8)	1.33	1.13, 1.56
	20−	682	18.2(13.6)	1.05	0.94, 1.17
	30−	1,002	16.8(13.1)	0.97	0.87, 1.08
	40−	979	16.6(13.5)	0.97	0.87, 1.08
	50−	822	12.7(10.3)	0.77	0.69, 0.86
	60−	756	12.0(10.0)	0.74	0.66, 0.83
	70+	515	10.4(9.4)	0.65	0.58, 0.73
Sex	Female	3,026	16.4(14.0)	1.00	
	Male	2,792	16.9(14.2)	1.01	0.97, 1.05
Season of survey	Summer	1,299	17.1(15.3)	1.00	
	Winter	4,519	16.5(13.7)	0.93	0.88, 0.98
Household size	1	86	12.2(12.1)	1.00	
	2	448	13.7(12.4)	1.10	0.91, 1.32
	3	1,930	16.9(14.1)	1.05	0.88, 1.25
	4	1,370	17.2(14.2)	1.13	0.95, 1.34
	5	886	16.3(13.8)	1.13	0.95, 1.35
	6+	1,098	17.2(14.6)	1.20	1.01, 1.43
Occupation	Employed	2,729	16.6(13.1)	1.00	
	Under education	878	26.2(18.0)	1.10	0.97, 1.24
	Unemployed	2,211	12.9(11.4)	0.94	0.88, 1.00
City	Foshan	2,154	14.9(11.1)	1.00	
	Guangzhou	2,666	16.7(15.9)	1.09	1.04, 1.15
	Zhuhai	998	20.4(13.8)	1.23	1.15, 1.3

Abbreviations: SPCs, supplementary professional contacts; SD, standard deviation

### Number of contacts

**Figure 1:**
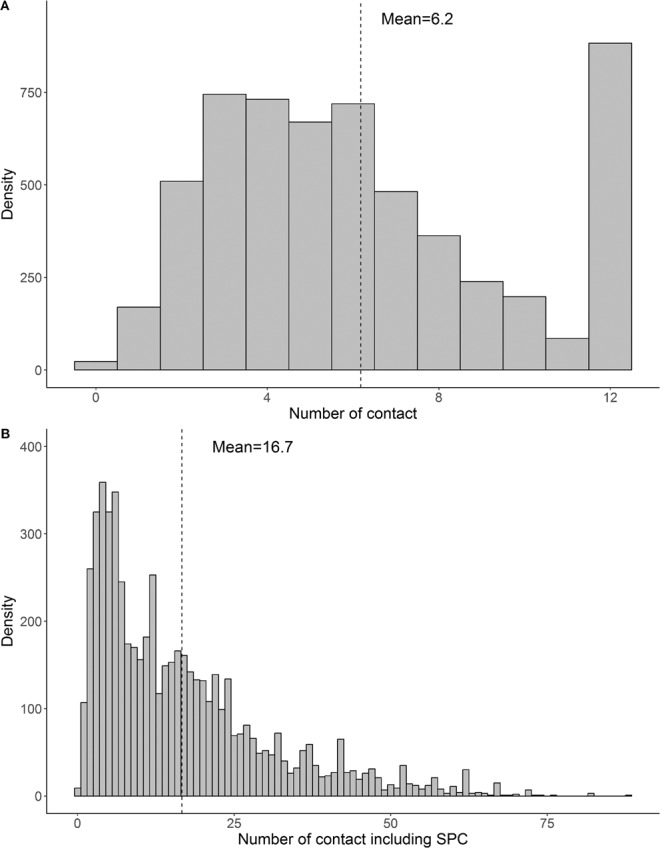
Distribution of contact number (Panel: **A**) and those including supplementary professional contacts (SPCs) (Panel: **B**), Guangdong, China, 2016. Maximum recorded items at 12 contacts per day (**A**). Abbreviations: SPCs, supplementary professional contacts.

As shown in Fig. 1A, 35,542 contacts were recorded, averaging 6.2 per day (SD, 3.3). The peak/maximum number of contacts was 12 for each participant (12: peak values, Fig. 1). When including SPCs, the number of contacts showed a fat-tail distribution and the average number of contacts for each participant per day was 16.7 (SD, 14.1), (Fig. 1B).

Table 1 shows no significant associations between sex, occupation and the number of contacts. However, age, survey season, city and household size over 6 were all related to the number of contacts. Compared to the 0–4 age group, 5–19 age groups had more contacts, whilst over 50 age groups had fewer contacts. No differences were observed amongst the 0–4 age group and 20–49 age group. In the summer, the average number of contacts was 17.1 (SD, 15.3), which was slightly higher than that of the winter season (mean, 16.5; SD, 13.7). Regarding cities, the mean number of contacts in Foshan were 14.9 (SD, 11.1), which was less than that of Guangzhou 16.7 (SD, 15.9) and Zhuhai 20.4 (SD, 13.8).

### Temporal distribution of the contact characteristics

As shown in Table 2, nearly half of the contacts (48.5%) occurred between relatives. In the summer, 40.8% of the contacts were relatives and nearly 30% were colleagues or classmates. In winter, the percentage of contacts with relatives increased to 51.7% and the number of colleagues or classmates decreased to 25.0%.

**Table 2:** Distribution of Contacted Persons according to Contact Features across the Different Seasons.

Category	Covariate	Frequency in a year (%)	Frequency in Summer (%)	Frequency in Winter (%)	*P*-value
Relationships	Relative	17,240(48.5)	4,284(40.8)	12,956(51.7)	<0.001
	Colleague/Schoolmate	9,365(26.3)	3,102(29.6)	6,263(25)	
	Friend	6,309(17.8)	1,660(15.8)	4,649(18.6)	
	Others	2,628(7.4)	1,449(13.8)	1,179(4.7)	
Location	Home	17,007(47.9)	4,140(39.4)	12,867(51.4)	<0.001
	School	3,322(9.3)	1,186(11.3)	2,136(8.5)	
	Office	6,190(17.4)	2,041(19.4)	4,149(16.6)	
	Transport	532(1.5)	167(1.6)	365(1.5)	
	Leisure	6,841(19.2)	2274(21.7)	4,567(18.2)	
	Others	2,263(6.4)	1,084(10.3)	1,179(4.7)	
Frequency	(Almost) daily	24,440(68.8)	7,350(70)	17,090(68.2)	<0.001
	Once-twice/week	7,269(20.5)	1,920(18.3)	5,349(21.4)	
	Once-twice/month	2,712(7.6)	799(7.6)	1,913(7.6)	
	Monthly	737(2.1)	206(2.0)	531(2.1)	
	First time	384(1.1)	220(2.1)	164(0.7)	
Duration	<5 min	1,298(3.7)	744(7.1)	554(2.2)	<0.001
	5–15 min	1,907(5.4)	790(7.5)	1,117(4.5)	
	15min-1hr	4,551(12.8)	1,198(11.4)	3,353(13.4)	
	1–4 hr	8,327(23.4)	1,929(18.4)	6,398(25.5)	
	>4 hr	19,459(54.7)	5,834(55.6)	13,625(54.4)	
Nature of contacts	physical	18,216(51.3)	3,226(30.7)	14,990(59.8)	<0.001
	non-physical	17,326(48.7)	7,273(69.3)	10,057(40.2)	

Abbreviations: hr, hours; min, minutes.

Homes had the highest number of contacts (47.9%), followed by leisure areas (19.2%), offices (17.4%), and schools (9.3%). In the summer, 40% of contacts occurred at home, which increased to 51.4% in the winter.

A total of 68.8% of the contacts occurred on a daily basis, but only 1.1% occurred for the first time. The proportion of contacts who met daily or on the first occasion in summer were slightly higher than in winter. The percentage of contacts who met 1–2 times per week in summer were lower than those in winter (18.3% vs 21.4%).

Approximately 54.7% of contacts lasted more than 4 hours and only 3.7% occurred for less than 5 minutes. The differences between summer and winter for contacts that lasted over 4 hours were small, but the proportion of contacts that lasted 1–4 hours in the winter were higher than those of the summer (25.5% vs 18.4%).

**Figure 2:**
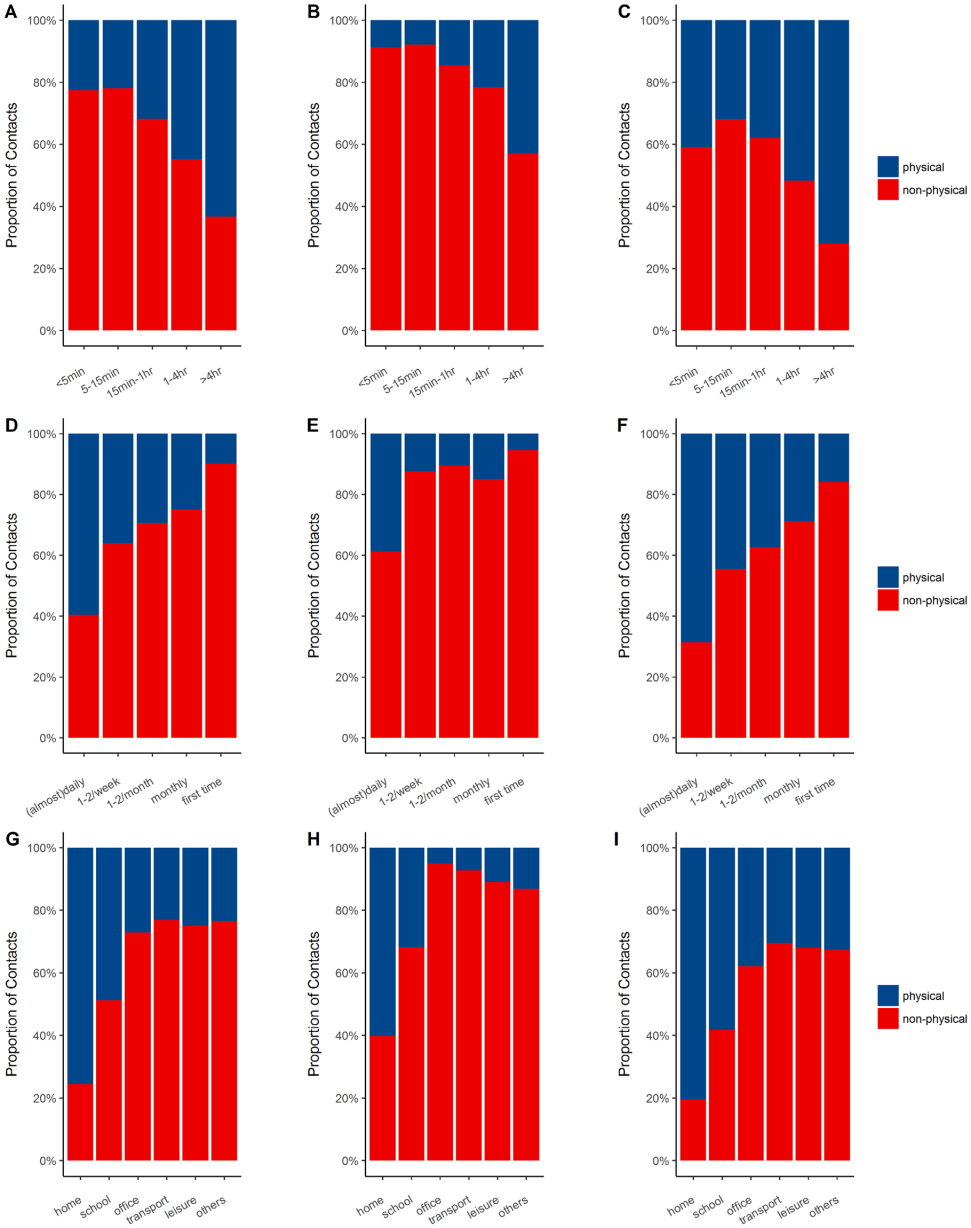
Percentage of physical or non-physical contacts by duration, frequency, and location per year (Panel: A, D, and G), summer (Panel: B, E, and H) and winter (Panel: C, F, and I). Abbreviations: hr, hours; min, minutes.

Approximately 51.3% of contacts were physical and the percentage in the summer (30.7%) was lower than the winter (59.8%). Figure 2 illustrates the percentage of contacts involving physical or non-physical contact according to duration, frequency, and location across the different seasons. Regarding duration, 63.3% of the contacts lasted over 4 hours and were physical, whilst the number of contacts in the winter (72.1%) were larger than the summer (42.9%). Regarding frequency, 59.6% of the contacts that met on a daily basis were physical, with contacts in the winter most frequently involving physical contact. Regarding location, physical contacts were most frequent at home (75.6%) and the percentage of physical contacts across each location in the summer were significantly lower than those in the winter, particularly for offices (5.1% in summer vs. 37.9% in winter).

**Figure 3:**
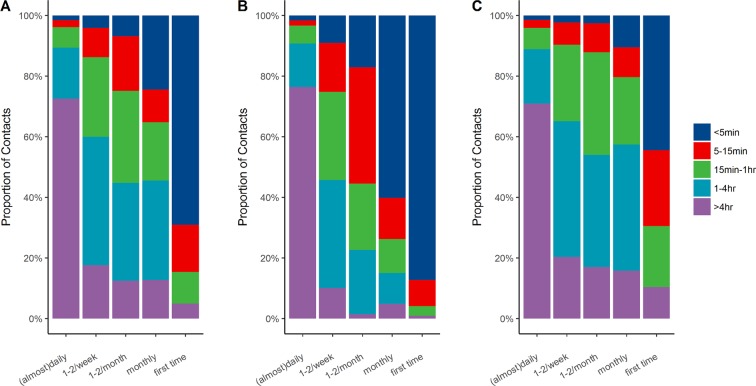
Percentage of contact duration according to frequency in all seasons (Panel: **A**), summer (Panel: **B**) and winter (Panel: **C**). Abbreviations: hr, hours; min, minutes.

Figure 3 shows that 72.5% of the contacts that occurred on each day lasted over 4 hours. Compared to winter, contacts in the summer were of a shorter duration and lower frequency.

### Spatial distribution of the contacts

Table 3 shows the different distributions of contacted individuals according to cities, the patterns of which differed (*P* < 0.001) but followed common characteristics. For example, the mostly frequently contacted individuals were relatives and the location with the highest number of contacts were households for each city. Moreover, the largest proportion of contact frequencies and duration were those that occurred daily and lasted more than 4 hours, respectively. The contacts occurring on daily basis lasted over 4 hours and those at home were more likely to involve physical contact in all 3 cities (S1 Fig). The percentage of contacts that were physical in Guangzhou (38.8%) were lower than those in Foshan (63.8%) and Zhuhai (76.0%).

**Table 3:** Spatial Distribution of the Contact Characteristics across the Different Cities.

Category	Covariate	Frequency in Foshan (%)	Frequency in Guangzhou (%)	Frequency in Zhuhai (%)	*P*-value
Relationships	Relative	6,621(57.2)	8,356(42.1)	2,263(55.1)	<0.001
	Colleague/Schoolmate	2,669(23.1)	5,453(27.5)	1,243(30.3)	
	Friend	2,002(17.3)	3,842(19.3)	465(11.3)	
	Others	283(2.4)	2,207(11.1)	138(3.4)	
Location	Home	6,679(57.7)	8,057(40.6)	2,271(55.3)	<0.001
	School	874(7.6)	2,045(10.3)	403(9.8)	
	Office	1,689(14.6)	3,611(18.2)	890(21.7)	
	Transport	117(1.0)	406(2.0)	9(0.2)	
	Leisure	1,783(15.4)	4,660(23.5)	398(9.7)	
	Others	561(4.8)	1,545(7.8)	157(3.8)	
Frequency	(Almost) daily	7,292(63.0)	13,859(69.8)	3,289(80.0)	<0.001
	Once-twice/week	2,668(23.0)	4,013(20.2)	588(14.3)	
	Once-twice/month	1,244(10.7)	1,264(6.4)	204(5.0)	
	Monthly	325(2.8)	398(2.0)	14(0.3)	
	First time	46(0.4)	324(1.6)	14(0.3)	
Duration	<5 min	161(1.4)	1,097(5.5)	40(1.0)	<0.001
	5–15 min	362(3.1)	1,419(7.1)	126(3.1)	
	15 min–1 hr	1,214(10.5)	2,912(14.7)	425(10.3)	
	1–4 hr	2,648(22.9)	5,174(26.1)	505(12.3)	
	>4 hr	7,190(62.1)	9,256(46.6)	3,013(73.3)	
Nature of contacts	physical	7,387(63.8)	7,706(38.8)	3,123(76.0)	<0.001
	non-physical	4,188(36.2)	12,152(61.2)	986(24.0)	

Abbreviations: hr, hours; min, minutes.

### Social mixing patterns according to age

**Figure 4:**
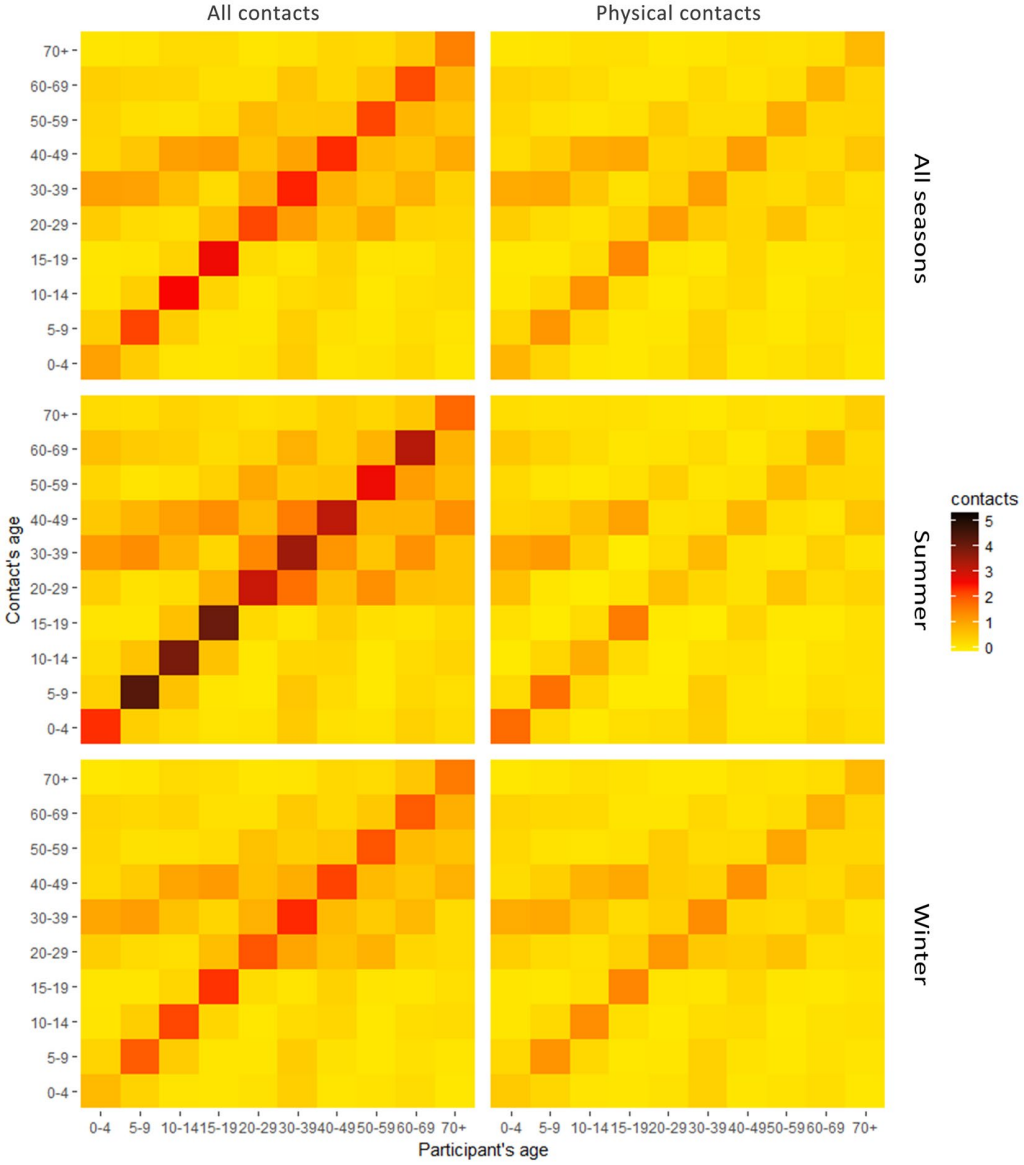
Contact intensity matrices of all contacts and physical contacts in the whole year, summer and winter.

Figure 4 shows the average number of contacts per person per day according to age groups for all contacts across all seasons. The contact matrix revealed that the diagonal element strengths were highest, meaning that all age groups tended to contact their peers. The two medium contact intensities were between 0–9 years and 30–39 years and 10–19 and 40–49 age groups. The contact intensity for the 30–49 age group plateaued, meaning that all age groups tended to get along with those aged 30–49 years old.

Some differences across the seasons were observed (Fig. 4). For all contacts, the diagnostic element strength in the summer was most remarkable. For physical contacts, the diagonal element strength in the winter was slightly stronger than that of the summer, except for 0–4 and 5–9 age groups. A non-symmetrized matrix was also performed (Supplementary Fig. S3).

## Discussion

Social contact refers to the connections between crowds that occur during daily routines. When an infectious disease is transmitted by airborne droplets or close contact in a susceptible population, the social contact patterns of the population influence the epidemic trend. In the past, social contact parameters based on assumptions were used to modeling infectious diseases, but recent studies suggest that actual social contact parameters obtained through surveys can help develop more accurate mathematical models for diseases such as influenza, mumps, chickenpox, parvovirus, and hand-foot-and-mouth disease^6,22–24^. Apart from the general characteristics, social contact patterns across different regions show various characteristics due to the economy, customs, cultural background, and population densities. Moreover, seasonal differences in contact patterns may provide clues to our understanding of the seasonality of infectious diseases. To-date, few large-scale contact pattern surveys^25^ have been performed to consider the seasonal differences of contact patterns. To develop more precise models of infectious disease transmission in mainland China, we performed a large-scale survey in the Guangdong Province of South China according to the POLYMOD study^1^ in Europe.

We found that the average number of contacts per person per day was 6.2 (16.7 with SPC). The numbers without SPC were similar to those reported in Vietnam (7.7)^11^, but lower than reported in Europe (13.4)^1^ and Taiwan (12.5)^7^. Students aged 10 to 14 years had the highest number of contacts, which resulted from intense contacts with schoolmates. The number of contacts were related to age, season, city, and household size, meaning that in addition to age-related contact patterns, spatiotemporal heterogeneity also occurred.

As previously reported^2,11,12,29,30^, the most common sites of contact were homes, which had the highest proportion of physical contact, which was comparable to POLYMOD^1^ and HongKong^31^. The next most common sites of contact were leisure areas, but only 27.4% of contacts were physical, consistent with that reported in Taiwan^7^ (27%) and Vietnam^11^ (10%), but lower than the reported values in the POLYMOD^1^ (50%). These findings indicate that the intimacy at home for Chinese individuals was similar to that of Europe. In the leisure areas, the Chinese were relatively reserved. This explains the cohort of infected individuals when a disease is transmitted by close contact in China, in which family members are primarily infected and the distribution of cases shows family clustering^32,33^. Thus, when preventing disease spread, close contact in family homes should be avoided.

Our study showed minimal differences in the number of contacts between summer and winter, with averages of 17.1 (SD,15.3) and 16.5 (SD,13.7) individuals, respectively, which was consistent to the study which compared the social contact patterns during flu season and non-flu season^25^. However, relationships, location, duration and the nature of social contacts show seasonal variations. In terms of the relationships and locations of the contacts, proportions of the contacts occurred at home and within relatives were both higher in winter than that in summer. We speculate that the summer is suitable for outdoor activities, so the majority spend time outdoors, limiting home contact. In contrast, people tend to warm themselves by staying at home in winter which increases the possibility of contacts at home. A study suggested that people spent more time every day on average indoors in cold weather^34^. In addition, the continuous recirculation of air indoors due to closing windows and doors to reduce the cold provides ideal conditions for virus transmission^27^. Secondly, contacts in winter are prolonged and involve a larger proportion of physical contacts compared to the summer, which may lead to an increased chance of virus transmission such as Enterovirus 71. In addition, characteristics including a long duration, high frequency and a larger proportion of physical and household contacts were generally accompanied. These findings help explain the long-standing hypothesis that responses to small changes in contact rates in the summer may lead to an epidemic^35^ in winter due to a higher number of indoor activities.

We found that the contact rates amongst those of a similar age were the highest, followed by inter-generational contacts, presumably parents and children. These findings were consistent with previous studies^1,2,22,36–38^, which signified that social contact patterns amongst those in different regions show similarities. Furthermore, we found that adults aged 30–49 had high and varied contact rates, which were also observed in Hong Kong (41–65yearsold)^31^ and Vietnam^11^ (26–65 years old) studies. These findings have significant public health implications. For example, when a novel infectious disease transmitted by air or close contact is prevalent in Guangdong, all age groups are susceptible and those aged 5–19 may be the most contagious due to their high contact rates^1^. In addition, the 30–49 age group had wide contact with other age groups and share a high risk of infection. Compared to winter, all contacts in the summer increased, but physical contact decreased, inferring that infectious diseases transmitted by air would be transmitted only by close contact in the summer.

This study had several strengths. The survey had a wide coverage and large sample size, which captured the characteristics of social contact patterns in the Pearl River Delta of Guangdong. Secondly, we performed surveys based on face-to-face interviews through trained interviewees, which may reduce the bias of the diary methods. Thirdly, we explored the spatiotemporal variation of social contacts, particularly the diversities of social contact patterns in different seasons, which were critical for infectious disease modeling.

However, several limitations should not be ignored. First, our study was retrospective because respondents were requested to recall their contacts without advanced notification or instruction in the face-to-face interviews. Compared to prospective design, the underreporting of contacts might exist due to recall bias^4,29,39,40^. Secondly, the survey only recorded 12 contacts items in the questionnaire, although extra records for supplementary professional contacts were produced, resulting in slight deviations. It was this difficult to determine whether all contacts above the 12 were work-related. Thirdly, we only captured contacts involving conversations, physical contact, and non-direct or short-term exposure^41^. Other methods that could transmit infectious diseases were not considered.

## Conclusions

In conclusion, this study comprehensively investigated the characteristics of social contact patterns and spatiotemporal distribution in the Pearl River Delta of Guangdong, which can provide specific contact parameters in developing infectious diseases models and improve the prediction accuracy of mathematical models for the prevention and control of infectious diseases.

### Ethical approval

The study was approved by the Institutional Review Board of Guangdong Provincial Center for Disease Control and Prevention. All methods were performed in accordance with relevant guidelines and regulations. Informed consent was obtained from all participants and/or their legal guardians.

## Supplementary information

Supplementary information

## Data Availability

Additional data have been provided as electronic supplementary material. Detailed data at the individual-level are available through the corresponding author Wenjun Ma (mawj@gdiph.org.cn).
