# Supplementary material for: Spatiotemporal heterogeneity of social contact patterns related to infectious diseases in the Guangdong Province, China
Source: Sci Rep. 2020 Apr 15;10:6119. doi: 10.1038/s41598-020-63383-z (PMC7160103; doi:10.1038/s41598-020-63383-z)
Supplement: Supplementary file 1 — Supplementary information [file 41598_2020_63383_MOESM1_ESM.pdf]

Supplementary Information

Spatiotemporal heterogeneity of social contact patterns related to infectious diseases in the Guangdong Province, China

Yulin Huang[1,2], Xiaoshuang Cai[3], Bing Zhang[2], Guanghu Zhu[2], Tao Liu[2], Pi Guo[4], Jianpeng Xiao[2], Xing Li[2], Weilin Zeng[2], Jianxiong Hu[2], Wenjun Ma[2,*]

[1.] The First Affiliated Hospital of Jinan University, Guangzhou, China

[2] Guangdong Provincial Institute of Public Health, Guangdong Provincial Center for Disease Control and Prevention, Guangzhou, China

[3] School of Basic Medicine, Jinan University, Guangzhou, China

[4] Medical College, Shantou University, Shantou, China

Figure S1.

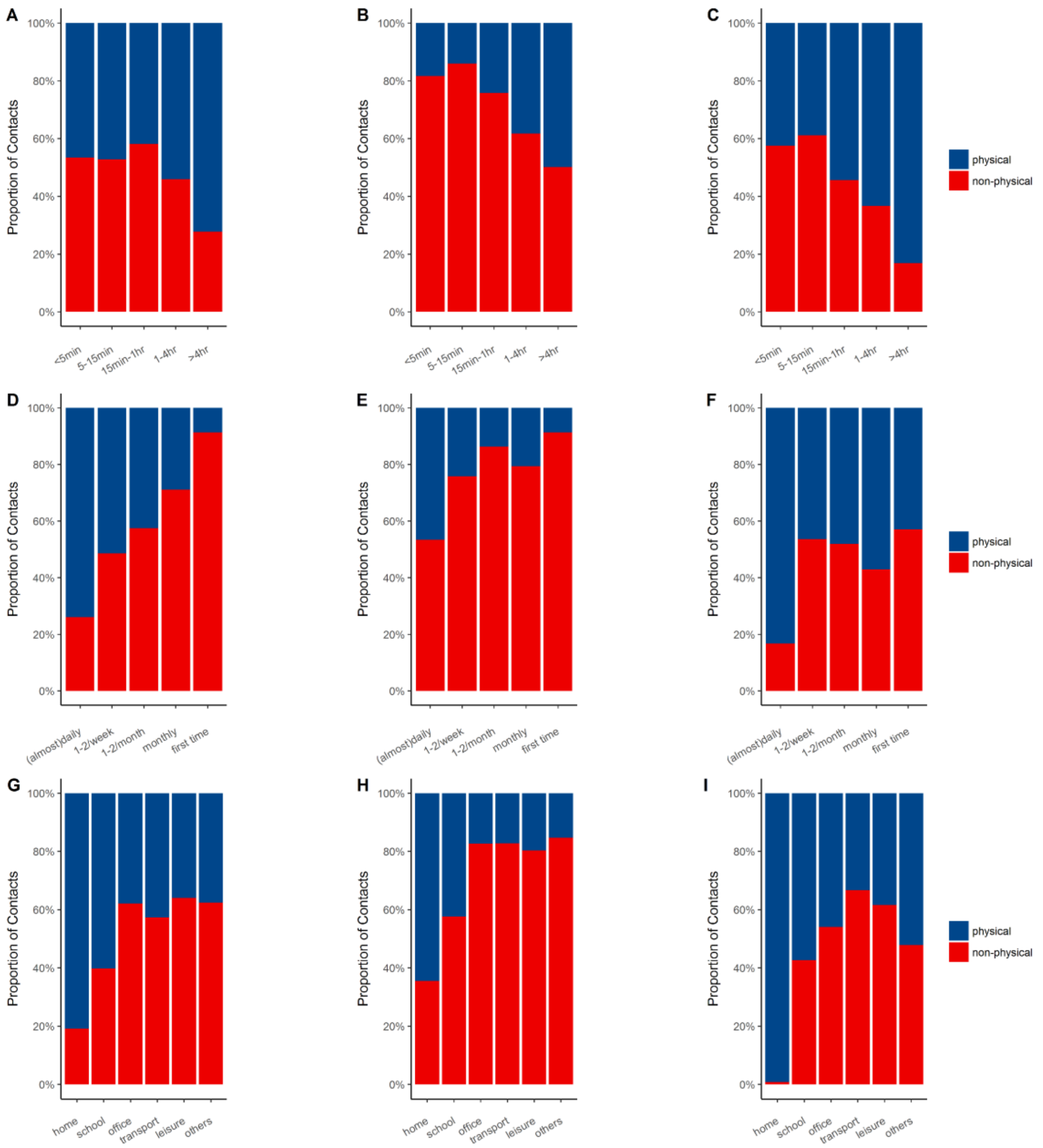

S1 Fig. Percentage of physical or non-physical contacts by duration, frequency, and location in Foshan (Panel: A, D, and G), Guangzhou (Panel: B, E, and H) and Zhuhai (Panel: C, F, and I) of Guangdong China. Abbreviations: hr, hours; min, minutes

Figure S2.

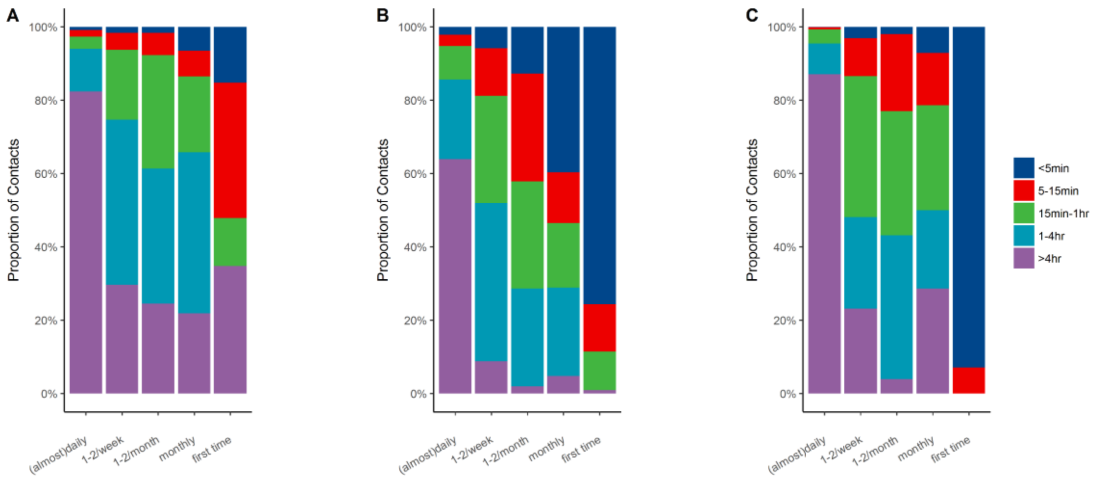

S2 Fig. Percentage of contact duration according to frequency in Foshan (A), Guangzhou (B) and Zhuhai(C) of Guangdong China. Abbreviations: hr, hours; min, minutes

Figure S3. Non-symmetrized contact intensity matrices of all contacts and physical contacts across the entire year, summer and winter.

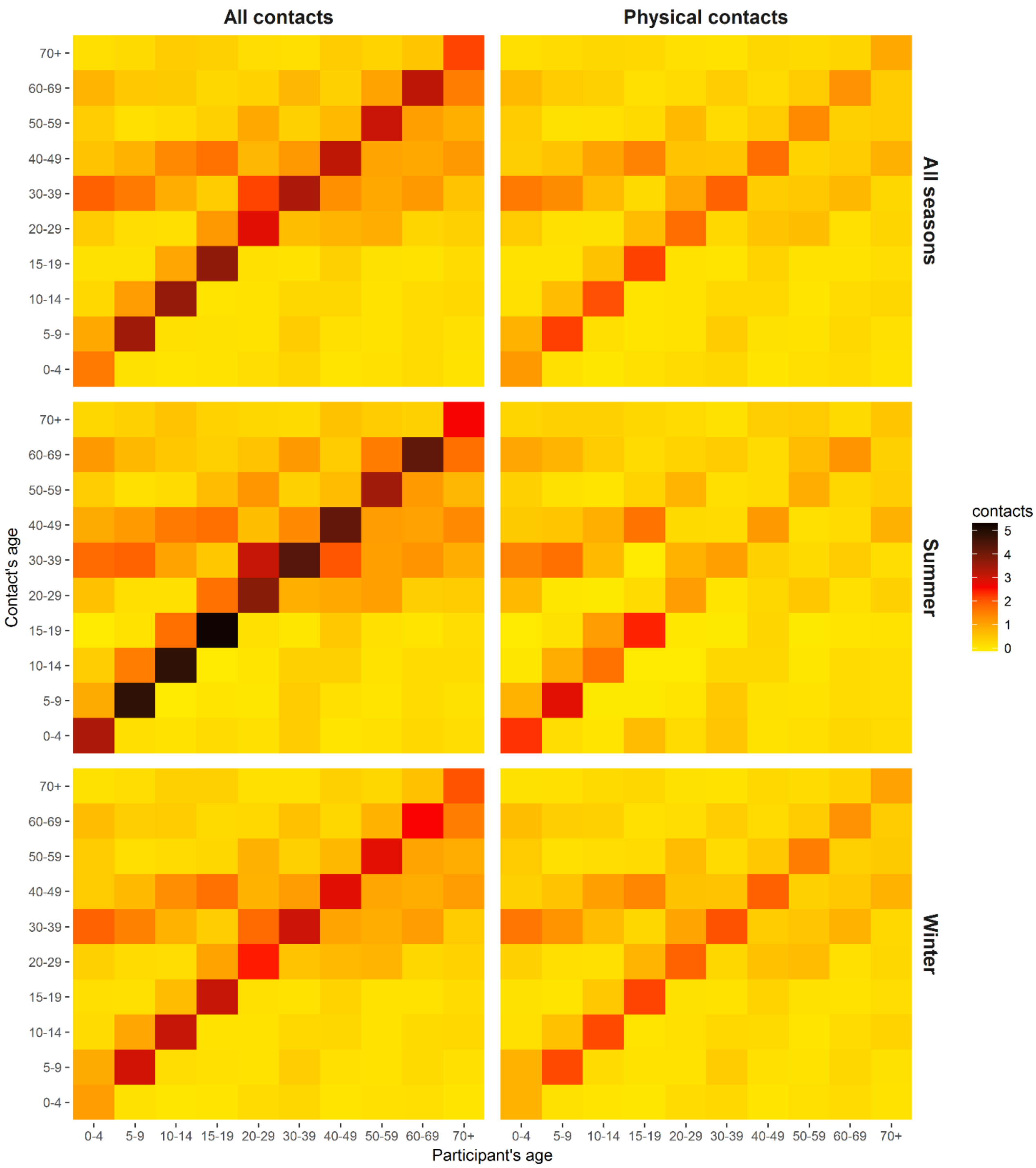

Supplementary Table S1. Comparison of age structure between census data (2015) and samples of Guangdong Province.

	Sample		Census		Ratio C/S
Age	N	%	N	%	Sampling weights
0-	301	5.17%	5624736	5.39%	1.043
5-	310	5.33%	5167116	4.95%	0.929
10-	246	4.23%	6812178	6.53%	1.544
15-	205	3.52%	9989559	9.58%	2.720
20-	682	11.72%	22896757	21.95%	1.873
30-	1,002	17.22%	18468133	17.70%	1.028
40-	979	16.83%	15911579	15.25%	0.906
50-	822	14.13%	9298010	8.91%	0.631
60-	756	12.99%	5234040	5.02%	0.386
70-	515	8.85%	4918351	4.71%	0.533
All	5818	100%	104320459	100%	1.000

Supplementary Text S1. Study questionare.

Survey ID:□□□□□□□□□□ Survey Date:

一、Demographic data

1.Name： ________

2.Gender： Male□ Female□

3.Age： _______ years

3.Household size（number of people living in your household）： ________

4.Occupation： _________

5 Address： __________________________________ Postcode：□□□□□□

二、Social contacts

Relationship				Age (or Range)	Gender		Physical Contact		Contact Frequency					location of contact						Total time spent with person during whole day				
Relatives	colleagues/ classmates	Friends	Others		Male	Female	Yes	No	almost every day	once or twice per week	once or twice per month	less than once per month	upon first meet	home	school	office	Transportation	leisure areas	other areas	less than 5mins	5- 15 mins	15 – 60 mins	1- 4 hrs	more than 4 hrs
□	□	□	□	□□(–	□	□	□	□	□	□	□	□	□	□	□	□	□	□	□	□	□	□	□	□

	□□)					
□ □ □ □	□□(− □□)	□ □	□ □	□ □ □ □ □	□ □ □ □ □ □	□ □ □ □ □
□ □ □ □	□□(− □□)	□ □	□ □	□ □ □ □ □	□ □ □ □ □ □	□ □ □ □ □
□ □ □ □	□□(− □□)	□ □	□ □	□ □ □ □ □	□ □ □ □ □ □	□ □ □ □ □
□ □ □ □	□□(− □□)	□ □	□ □	□ □ □ □ □	□ □ □ □ □ □	□ □ □ □ □
□ □ □ □	□□(− □□)	□ □	□ □	□ □ □ □ □	□ □ □ □ □ □	□ □ □ □ □
□ □ □ □	□□(− □□)	□ □	□ □	□ □ □ □ □	□ □ □ □ □ □	□ □ □ □ □
□ □ □ □	□□(− □□)	□ □	□ □	□ □ □ □ □	□ □ □ □ □ □	□ □ □ □ □
□ □ □ □	□□(− □□)	□ □	□ □	□ □ □ □ □	□ □ □ □ □ □	□ □ □ □ □
□ □ □ □	□□(− □□)	□ □	□ □	□ □ □ □ □	□ □ □ □ □ □	□ □ □ □ □
□ □ □ □	□□(− □□)	□ □	□ □	□ □ □ □ □	□ □ □ □ □ □	□ □ □ □ □
□ □ □ □	□□(− □□)	□ □	□ □	□ □ □ □ □	□ □ □ □ □ □	□ □ □ □ □
We do ask you to include every contact you had, but if you were unable to include every single contact (for instance, because you work in a shop till and have a large number of contacts in a day), please could you indicate this?						

☐I included every person I had contact with. ☐I did not include every person I had contact with.

If you did not include every person you had contact with, approximately how many people you left out did you have：

Physical contact with：________ No physical contact with：________

Instructions：

1. A contact is defined as：①EITHER a **two-way conversation** with three or more words in the physical presence of another person； ② OR physical **skin-to-skin contact** (for example a handshake, hug, kiss or contact sports).
2. The order in which you write down your contact persons is not important.
3. Contacts made exclusively by telephone or mobile phone should NOT be recorded.
4. If you contact the same person several times in the course of the day, only record him/her once, and record the total time you spent with that person over the entire day. So each person you meet during the day and have contact with should only have one line in the diary: one person, one line.。
5. If you don't know the exact age, give an estimate of the age range (e.g. 40-45) and try to make it as narrow as possible.。
6. Questionares for young children should be responded by a parent or guardian on their behalf.
